# Seeing the Forest and Its Trees Together: Implementing 3D Light Microscopy Pipelines for Cell Type Mapping in the Mouse Brain

**DOI:** 10.3389/fnana.2021.787601

**Published:** 2022-01-14

**Authors:** Kyra T. Newmaster, Fae A. Kronman, Yuan-ting Wu, Yongsoo Kim

**Affiliations:** Department of Neural and Behavioral Sciences, The Pennsylvania State University, Hershey, PA, United States

**Keywords:** brain mapping, cell type, serial two-photon tomography, light sheet fluorescence microscopy, data confederation, digital atlas, image analysis, image registration

## Abstract

The brain is composed of diverse neuronal and non-neuronal cell types with complex regional connectivity patterns that create the anatomical infrastructure underlying cognition. Remarkable advances in neuroscience techniques enable labeling and imaging of these individual cell types and their interactions throughout intact mammalian brains at a cellular resolution allowing neuroscientists to examine microscopic details in macroscopic brain circuits. Nevertheless, implementing these tools is fraught with many technical and analytical challenges with a need for high-level data analysis. Here we review key technical considerations for implementing a brain mapping pipeline using the mouse brain as a primary model system. Specifically, we provide practical details for choosing methods including cell type specific labeling, sample preparation (e.g., tissue clearing), microscopy modalities, image processing, and data analysis (e.g., image registration to standard atlases). We also highlight the need to develop better 3D atlases with standardized anatomical labels and nomenclature across species and developmental time points to extend the mapping to other species including humans and to facilitate data sharing, confederation, and integrative analysis. In summary, this review provides key elements and currently available resources to consider while developing and implementing high-resolution mapping methods.

## Introduction

Macro-circuits across the brain integrate information from regional micro-circuits to process external stimuli, evaluate internal state, and generate behavior (e.g., motor action). Each micro-circuit contains a diverse array of neuronal and non-neuronal cell types with region- specific compositions. For example, glutamatergic (excitatory) and GABAergic (inhibitory) neurons are the two major neocortical neuron types with a respective ratio of 4:1 in the mouse brain (Isaacson and Scanziani, 2011; Tremblay et al., 2016). Moreover, recent single cell transcriptomic approaches combined with other physiological analyses unveiled that more than 100 different neuronal cell types exist in the mouse isocortex (neocortex) with distinct connectivity, physiology, and molecular characteristics (Belgard et al., 2011; Zeng et al., 2012; Fuzik et al., 2016; Tasic et al., 2016, 2018; Tremblay et al., 2016; Zeng and Sanes, 2017; Lim et al., 2018; Saunders et al., 2018; Zeisel et al., 2018; Kanari et al., 2019; Winnubst et al., 2019; Gouwens et al., 2020; Ortiz et al., 2020; Scala et al., 2020; Yao et al., 2021). Notably, different functional regions within the neocortex such as the primary motor-sensory and association cortices showed different densities of excitatory and inhibitory neurons as well as variation in their cerebrovascular networks (Kim et al., 2017; Yun et al., 2019; Wu et al., 2021). Investigation of long–range connectivity within the neocortex and closely connected brain regions also identified modular and integrative circuits across brain regions (Oh et al., 2014; Zingg et al., 2014; Hintiryan et al., 2016; Harris et al., 2019; Foster et al., 2020). Unveiling the spatial distribution of cell types throughout the cortex provides useful information for inferring how the cortex and its subdivisions may function. Likewise, comprehensive knowledge of cell type distribution throughout the whole brain is critical to fully understanding the dynamic intricacies and exceptional breadth of brain function.

To visualize and quantify the spatial distribution of cell types in the entire brain, high-resolution imaging across the whole 3D volume is necessary. Microscopy techniques can provide up to submicron resolution details, but in its original design, microscopy does not lend itself to volumetric imaging. Conversely, many neuroimaging tools such as magnetic resonance imaging (MRI) can provide volumetric imaging, but they don’t offer sufficient resolution to image individual cells and lack the ability to distinguish individual cell types. Mesoscale imaging bridges these two scales by combining high-resolution 3D imaging and computational analysis to allow visualization and quantification of axons, cell nuclei, processes, and even synapses in intact biological samples (Odgaard et al., 1990; Dodt et al., 2007; Mayerich et al., 2008; Li et al., 2010; Zheng et al., 2019; Ueda et al., 2020a, b). This technical breakthrough has ushered in a new era of neuroanatomy providing new opportunities to advance our knowledge of the principles governing nervous system organization and function.

**Figure 1:**
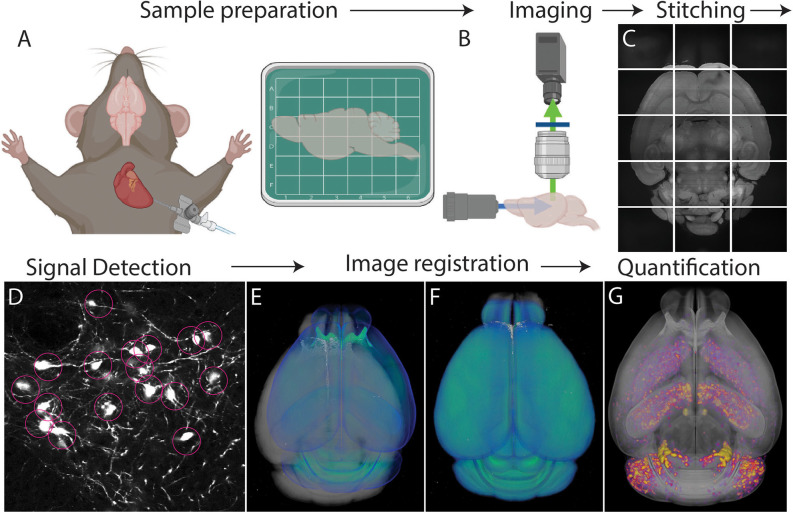
Whole brain cell type mapping pipeline. **(A)** Perfusion and fixation of the brain stabilize target signals. Fixed brains labeled with fluorescent signals can be further processed to lower background using tissue clearing. **(B)** Tiled images are acquired throughout the entire brain using whole brain 3D microscopy. **(C)** The resulting images are stitched to reconstruct the 3D volume of the brain. **(D)** Computational algorithms detect target signals automatically throughout the brain. **(E,F)** An individual brain (gray) is registered to an annotated standardized atlas (blue). **(E)** before and **(F)** after the registration. **(G)** Detected signals (purple/yellow) are mapped onto the atlas that allows either region of interest (ROI) or evenly spaced voxel-based analysis throughout the whole brain.

Although 3D cell type mapping is highly useful, it comes with technical and analytical challenges that require a systematic workflow (Figure 1). Thus, the goal of this review is to provide insight into how scientists can acquire and utilize different forms of brain mapping to precisely examine their biological questions. Many previous works have focused on the mouse brain due to the variety of available transgenic animals, the compact size of the mouse brain, and the availability of high-quality 3D atlases. However, similar mapping approaches are also possible in other species such as the non-human primate (NHP) as well as human brains (Woodward et al., 2020; Rapan et al., 2021; Shapson-Coe et al., 2021).

### 1. Cell Type Specific Labeling

**Figure 2:**
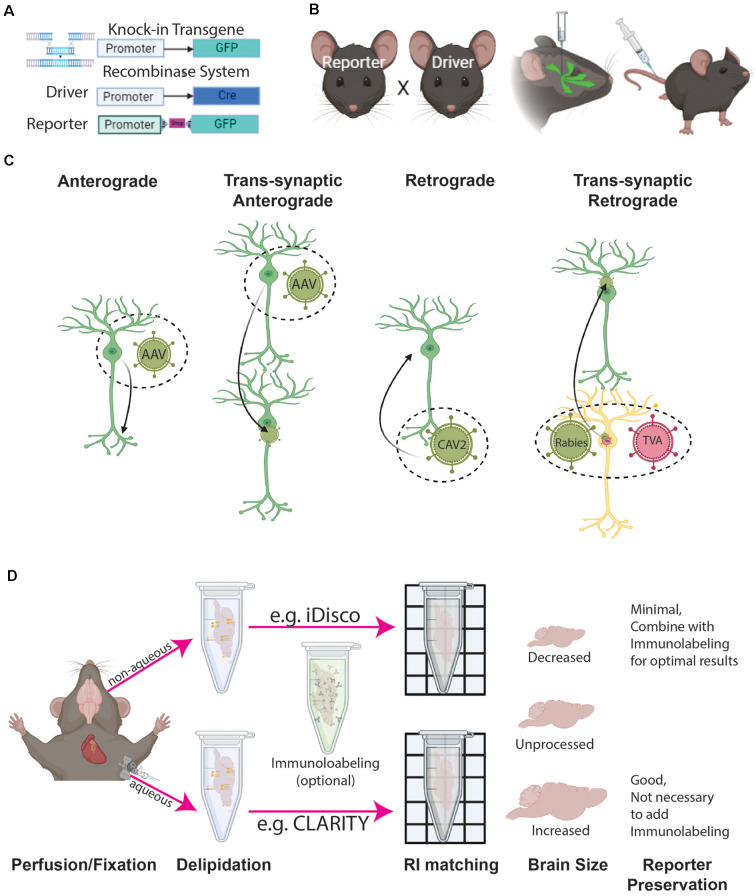
Cell type labeling and tissue clearing methods. **(A)** Genetic labeling methods include either reporter genes (e.g., GFP) directedly driven by the cell type specific promoter, or recombinase systems driven by a combination of cell type specific recombinase (e.g., Cre) expression and conditional (e.g., LoxP-Stop-LoxP) reporter gene expression (e.g., GFP). **(B)** Labeling can be achieved by the breeding reporter and driver animals and/or combining them with viral tools injected either directly into a target brain location or peripheral areas (e.g., tail vein). **(C)** Different configurations of viral tools provide ways to label specific cell compartments and their connection with other cells. **(D)** 3D immunohistochemistry with tissue clearing can be achieved by either aqueous or non-aqueous methods, both of which affect overall tissue volume.

In order to assess the distribution of cell types across the brain, genetic or histological methods can be used to distinguish specific cells from background information (Figure 2). Genetic methods leverage cell type specific promoters to drive reporter expression directly or indirectly through transgenic animals, viral administration, or a combination of both (Madisen et al., 2010; Josh Huang and Zeng, 2013; Daigle et al., 2018; Newmaster et al., 2020). Histological labeling is achieved through whole brain 3D immunohistochemistry with cell type specific antibodies and various tissue clearing methods (Renier et al., 2014; Yun et al., 2019; Kirst et al., 2020; Ueda et al., 2020b). Each labeling method has both benefits and limitations, making it important to choose a method that best suits the proposed experiment.

#### 1.1. Transgenic Animals

Genetic labeling with transgenic animals provides a way to examine different cell populations across the whole brain without immunohistochemistry (Luo et al., 2008; Taniguchi et al., 2011; Josh Huang and Zeng, 2013; Figure 2A). One of the most popular approaches is the Cre recombinase system. In this system, Cre recombinase is expressed from a knock-in locus that is driven by a cell type specific promoter (Gong et al., 2007; Madisen et al., 2010; Harris et al., 2014). The knock-in approach labels target cells with high accuracy and can be combined with various conditional reporter animals to express different reporter proteins making the Cre system highly flexible (Madisen et al., 2010; Taniguchi et al., 2011; Harris et al., 2014; Madisen et al., 2015; Daigle et al., 2018; Figure 2A). Reporter variants include several different colors (e.g., Ai14 with tdTomato, Ai75 with nuclear tdTomato, or Ai140 with an extremely high-level of GFP), Ca^++^ reporters (e.g., Ai96 with gCaMP6 s), sparse labeling models (e.g., MORF), and tools to manipulate activity (e.g., CAG-LSL-Gq-DREADD, Ai32 with channelrhodopsin 2; Gong et al., 2007; Taniguchi et al., 2011; Madisen et al., 2012, 2015; Harris et al., 2014; Zhu et al., 2016; Veldman et al., 2020).

To examine the overall distribution of target cells throughout the whole brain, a Cre driver animal can be bred with a conditional reporter animal which leads to offspring with the desired cell type labeled (Harris et al., 2014; Figure 2B). If the goal is to examine a specific cell type in an anatomically defined region, intracerebral injection of a virus carrying a reporter or Cre recombinase will provide spatial specificity in addition to cell type specificity (see the next section for more details; Josh Huang and Zeng, 2013; He et al., 2016; Jeong et al., 2016; Son et al., 2020; Figure 2B). Flippase (flp), another popular recombinase, can be combined with Cre to further identify subtypes within a cell type population (Farago et al., 2006; Miyoshi et al., 2010; Taniguchi et al., 2011; Josh Huang and Zeng, 2013; He et al., 2016; Graybuck et al., 2020). Inducible recombinase (e.g., tamoxifen inducible CreERT) provides temporally restricted recombinase activity which is useful in the study of cell type development from a defined time point (Taniguchi et al., 2011; Wu et al., 2021). This type of recombinase can also be used to achieve sparse labeling by titrating recombinase activating drugs (e.g., tamoxifen). Sparse labeling is particularly useful for dense cell types and single cell tracing experiments. Another way to achieve sparse labeling is through the MORF mouse which was designed to stochastically label 1–3% of Cre expressing cells (Veldman et al., 2020).

One caveat of Cre recombinase-based labeling is its lack of temporal resolution. Animals derived from the pairing of a conditional reporter mouse and a Cre expressing mouse will express a fluorescent reporter after a single Cre activation even when the gene of interest and Cre are no longer being expressed. Therefore, these animals may not accurately represent temporal patterns of gene expression (Song and Palmiter, 2018; Newmaster et al., 2020). This issue can be circumvented with the use of transgenic animals with direct reporter expression under specific cell type specific promoters. Regardless of reporter mice, it is important to validate the expression of reporter genes against the gene of choice using immunohistochemistry or mRNA *in situ* prior to mapping effort as transgenic animals may lead to misrepresent or over represent target cell type expression (Zoghbi, 2003; Gerfen et al., 2013; Newmaster et al., 2020).

More detailed information about different recombinase based cell type specific drivers and conditional reporter animals, reference the characterization databases can be found in Allen Institute for Brain Science^1^, the GENSAT project^2^, and the NIH Neuroscience Blueprint Cre Driver Network^3^. We also refer to recent review articles summarizing a different set of genetic tools to label specific cell types (Kim and Dymecki, 2009; Madisen et al., 2010; Taniguchi et al., 2011; Gerfen et al., 2013; Josh Huang and Zeng, 2013; Harris et al., 2014; McLellan et al., 2017; Daigle et al., 2018; Debbache et al., 2018; Yook et al., 2021).

#### 1.2. Viral Tools

Virus-mediated labeling has been critical to examine cell type distribution in anatomically defined areas and cell-type specific connectivity throughout the brain (Oh et al., 2014; He et al., 2016; Jeong et al., 2016; Harris et al., 2019; Figures 2B,C). To assess anterograde connectivity, adeno-associated virus (AAV) with a recombinase-dependent reporter can be injected into the target area of a cell-type specific Cre and/or Flp driver animal (Figure 2C). This approach has been widely used to establish wiring diagrams of different cell types in the mouse brain (Oh et al., 2014; Gerfen et al., 2018; Harris et al., 2019; Winnubst et al., 2019; Son et al., 2020). The main caveat to this approach is that only cellular processes (e.g., axons and axon terminals) are visualized and one cannot determine which cells in the targeted regions are receiving synaptic input. To address this, trans-synaptic anterograde tracing with different serotypes of AAV and herpes simplex virus is under development, but the robustness of these viruses in mono trans-synaptic anterograde tracing remains to be established (Lo and Anderson, 2011; Zingg et al., 2017; Cembrowski et al., 2018; Su et al., 2020; Zingg et al., 2020; Yook et al., 2021; Figure 2C). For retrograde tracing, viruses such as retro AAV or Canine adenovirus type 2 (CAV-2) can be injected into a region to be taken up by presynaptic terminals and transported to the cell nucleus for reporter expression (Junyent and Kremer, 2015; Tervo et al., 2016; Li et al., 2018; Figure 2C). For mono-synaptic retrograde tracing, rabies viruses coupled with helper viruses that restrict retrograde jumping to one neuron have been well-established and widely used (Callaway and Luo, 2015; Schwarz et al., 2015; Pomeranz et al., 2017; Son et al., 2020; Huang et al., 2021; Figure 2C).

Virus-mediated labeling has become even more flexible with the invention of AAV viruses packaged with plasmids that contain enhancers for cell type specific expression of reporter genes (Chan et al., 2017; Mich et al., 2021). When combined with blood-brain barrier penetrating viruses (e.g., AAVphp.eb), peripheral injection of this viral tool can achieve cell type specific labeling in any animal opening new paths to cell type mapping in non-traditional model animals and animal models of disease (Chan et al., 2017; Mich et al., 2021; Figure 2B). With so much flexibility, virus-mediated cell type labeling is a versatile tool that simply requires a little creativity to effectively use. We refer to recent excellent reviews that summarize the recent development and application of viral tools for more details and guidance on experimental design (Callaway and Luo, 2015; Bedbrook et al., 2018; Haery et al., 2019; Saleeba et al., 2019; Suzuki et al., 2020; Cong et al., 2020; Lanciego and Wouterlood, 2020; Nectow and Nestler, 2020).

#### 1.3. 3D Immunolabeling With Tissue Clearing

Remarkable advances in tissue clearing and 3D immunolabeling make it possible to label target cells with specific antibodies in intact biological samples. This enables scientists to bypass complex breeding schemes and care for excessively large colonies accelerating new discoveries and extending brain mapping technology to new species including humans (Lai et al., 2018; Kim et al., 2021). Here, we provide a brief summary of a few widely used clearing and chemical labeling methods to examine cell type distribution in the whole brain. Tissue clearing involves delipidation (first part of tissue clearing) and index matching (second part of the clearing) with an optional and often necessary immunolabeling step (Ueda et al., 2020a, b; Figure 2D). Tissue clearing can be aqueous or non-aqueous (Jensen and Berg, 2017; Qi et al., 2019; Yun et al., 2019; Ueda et al., 2020a, b; Figure 2D). Furthermore, full brain clearing and 3D immunolabeling can be achieved through active or passive methods. Active methods (e.g., CLARITY, SHIELD) use electrophoresis and chemical engineering to increase the homogeneity of clearing and immunolabeling (Chung et al., 2013; Chung and Deisseroth, 2013; Yun et al., 2019; Ueda et al., 2020a, b). However, the procedure is costly due to the required equipment and often only allows for the processing of small batches per round. Passive clearing (e.g., iDISCO) can take longer especially with additional immunolabeling, but it is less expensive and a large number of samples can be processed at the same time (Renier et al., 2014; Qi et al., 2019).

Different tissue clearing methods can introduce varying degrees of tissue deformation (Wan et al., 2018; Ueda et al., 2020a, b; Weiss et al., 2021). Aqueous methods tend to bloat the tissue which can introduce tearing of delicate areas and connected structures such as axons and vasculature (Chung et al., 2013; Wan et al., 2018). However, aqueous clearing methods can preserve native fluorescence well (Chung et al., 2013; Wan et al., 2018). In contrast, non-aqueous methods use tissue dehydration to finalize the clearing process which often causes tissue shrinkage (Wan et al., 2018). Moreover, non-aqueous clearing methods tend to quench endogenous fluorescent proteins (Renier et al., 2014; Qi et al., 2019). Recently developed non-aqueous methods such as FDISCO improve the preservation of endogenous fluorescence, but the native signals begin to fade after a few days, limiting the window of opportunity for sample imaging (Qi et al., 2019). This problem can be overcome by performing immunolabeling for endogenous reporter proteins with a more stable secondary antibody (Renier et al., 2014). An additional benefit of sample dehydration is that the tissue becomes plasticized, which makes handling delicate samples (e.g., early developing brains) easier and tissue shrinkage can facilitate imaging of larger tissue samples (Wan et al., 2018).

Finally, it is important to consider the experimental needs and location of the structure that is being targeted. Cell counting is best done with signals restricted to the cell nucleus or cell body. On the other hand, tracing and visualization of subcellular structures such as spines will require labeling that fills the entire cell or localizes to the cell membrane. Visualizing subcellular structures will also benefit from expansion seen in aqueous clearing methods (Matryba et al., 2019). In short, no one method will work for all antibodies or probes due to different biophysical properties of proteins and mRNA, so the appropriate literature searches and initial pilot testing should be done before choosing a particular method (Weiss et al., 2021). For an in-depth discussion of 3D tissue labeling and clearing please see: Molbay et al. (2021), Ueda et al. (2020a,b), and Weiss et al. (2021).

### 2. Imaging

**Figure 3:**
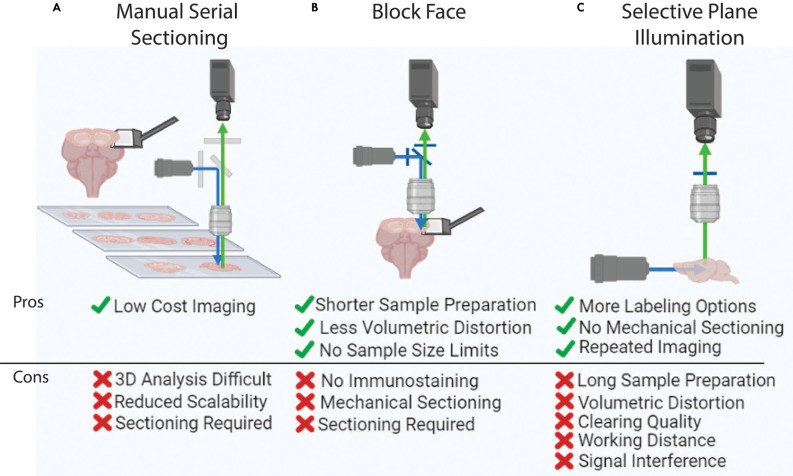
Whole brain imaging methods.** (A)** Manual cutting and imaging histological sections on slides. This method is laborious and requires significant amounts of manual effort. **(B)** Block-face imaging such as serial two photon tomography. The requirement of serial sectioning can introduce challenges in delicate samples. **(C)** Selective plane illumination or light-sheet imaging. This method can visualize an intact and clear sample without any physical sectioning, yet this imaging is hard to apply to large tissue (e.g., human brain) due to the limited working distance of an objective lens.

Following sample labeling and preparation, 3D imaging with sufficient resolution to examine individual cells and their processes in intact biological samples can be performed (Figure 3). The most basic yet labor-intensive imaging method is using serial sectioning and tiled microscopic imaging of 2D sections followed by 3D reconstruction (Zingg et al., 2014; Hintiryan et al., 2016; Zingg et al., 2018; Cizeron et al., 2020; Benavidez et al., 2021; Figure 3A). Though this method is readily available to most labs, it is difficult to scale up. High-speed slide scanners such as the Nanozoomer (Hamamatsu) can make this method more streamlined and have even been used to achieve brain mapping in many different species including the marmoset (Zheng et al., 2014; Lin et al., 2019; Woodward et al., 2020). However, histological sectioning and mounting on microscopic slides can also present a challenge as these procedures introduce unexpected volume distortion, making 3D reconstruction challenging. Here, we will review two commonly used automated 3D high-resolution imaging methods that ensure precise 3D reconstruction; block-face imaging and selective plane illumination microscopy (SPIM) imaging. Each system has unique advantages and disadvantages (Figure 3). Labeling type, structure of interest, and sample properties are the main dictators of which imaging modality to choose.

#### 2.1. Block-Face Imaging

Block-face imaging utilizes tiled microscopic images of the surface of an intact sample block followed by serial sectioning (Ragan et al., 2012; Zheng et al., 2013, 2019; Amato et al., 2016; Economo et al., 2016; Figure 3B). Serial two photon tomography (STPT) performs tiled block-face imaging with a multiphoton microscope and high fidelity vibratome with typical sectioning at either 50 μm or 100 μm thick (Oh et al., 2014; Kim et al., 2017; Newmaster et al., 2020). Finer z-resolution imaging can be acquired by adding optical sectioning within each slice (Wu et al., 2021). STPT is a flexible modality that has been used to map neural connectivity, cell-type density, cerebrovasculature, and dendritic architecture (Tsai et al., 2009; Oh et al., 2014; Zapiec and Mombaerts, 2015; Kim et al., 2017; Newmaster et al., 2020; Wu et al., 2021). However, it is important to note that STPT requires endogenous fluorescence expression of target signals. Therefore, STPT mapping heavily relies on transgenic reporter animals which can require complex breeding schemes (Amato et al., 2016; McLellan et al., 2017). However, the recent developments in viral tools such as AAVphp.eb and enhancer-based viral tools enable the expression of fluorescent proteins in non-transgenic animals discussed above, open new possibilities for expanding the implementation of STPT brain mapping (Chan et al., 2017; Mich et al., 2021). One distinct advantage of STPT is simple sample preparation (Figure 3B). Fixed brains can be used without further tissue clearing. Another advantage to this imaging method is that the working distance of the objective lens does not dictate the image depth of the system because serial sectioning removes the sample surface as imaging progresses through the volume. Therefore, STPT can be employed to examine larger samples such as the marmoset and human brain (Okano and Mitra, 2015; Abe et al., 2017; Lin et al., 2019; Mancini et al., 2020).

Knife-Edge Scanning Microscopy and Micro-optical sectioning tomography (MOST) are other variations of block-face imaging which were originally developed with light microscopy to achieve high-resolution 3D imaging with ultrathin sectioning (less than 1 μm; Mayerich et al., 2008; Zheng et al., 2013, 2019). Fluorescence MOST (fMOST) combines fluorescence microscopy with MOST to take advantage of cell type specific labeling. This technology images the sample on the edge of a diamond blade as sectioning occurs (Zheng et al., 2013, 2019; Figure 3B). To achieve thin sections, brain samples are embedded in resin or hard plastic. Thin sectioning along with a high numerical aperture oil lens improves imaging resolution allowing structures such as individual axons and dendritic spines to be captured for highly accurate axon tracing and neuron reconstruction experiments (Zheng et al., 2013, 2019). Moreover, a recently developed line-illumination modulation technique further improves signal-to-noise ratio and acquisition speed (Zhong et al., 2021). The major drawbacks of this system are that it is not commercially available and imaging at such a high-resolution often generates more than 10 TB of data for one mouse brain requiring a specialized data analysis infrastructure.

#### 2.2. SPIM

SPIM or light-sheet imaging generates tiled images by illuminating a plane of optically cleared tissue and collecting signals in the orthogonal direction (Corsetti et al., 2019; Figure 3C). Light-sheet imaging was originally developed over 100 years ago, yet it did not become popular until the recent advances in tissue clearing (Corsetti et al., 2019; Ueda et al., 2020a, b). The illumination sheet of light is generated *via* transverse facing lenses and the signal is then collected from a second objective lens which can have many positions but is typically in the orthogonal direction (Corsetti et al., 2019). To obtain full brain data, the entire sample is moved through the imaging field with a motorized stage which triggers a CMOS camera for image collection (Ueda et al., 2020a). Because there is no need to section the sample and the field of view (FOV) is large, SPIM has dramatically reduced imaging time compared to STPT and is ideal for delicate samples (e.g., developing embryos). SPIM also has the added benefit of being intrinsically paired with 3D immunolabeling and tissue clearing methods which can be easily applied to different biological samples with any genetic labeling strategy (Roostalu et al., 2019; Gómez et al., 2021).

Nonetheless, SPIM also has a number of complicated drawbacks stemming from tissue preparation and the physical properties of the illumination system (Wan et al., 2018; Xu et al., 2019; Ueda et al., 2020a, b; Molbay et al., 2021; Weiss et al., 2021). SPIM requires samples to be optically clear which is time-consuming and often introduces tissue distortion (Dodt et al., 2007; Renier et al., 2014; Jensen and Berg, 2017; Wan et al., 2018; Qi et al., 2019; Figure 3C). Clearing technology itself also limits the size of the sample as it is difficult to remove lipids/penetrate larger volumes such as those of non-human primates and human brains (Tainaka et al., 2018; Zhao et al., 2019; Ueda et al., 2020a; Figure 3C). Even if these brains could be cleared, it is challenging to create an objective lens with a long enough working distance to collect signals from the entire sample (Ueda et al., 2020a). Some trials are currently underway to combine slab sectioning and SPIM imaging to overcome this limitation (Hillman et al., 2019; Voleti et al., 2019). For a more in-depth discussion of SPIM, refer to the following review articles (Elisa et al., 2018; Müllenbroich et al., 2018; Wan et al., 2018; Corsetti et al., 2019; Hillman et al., 2019; Ueda et al., 2020a).

### 3. Image Pre-Processing and 3D Image Reconstruction (Stitching)

Before images can be analyzed in 3D, raw data must be preprocessed to compensate for image distortion in each image tile and re-assembled into a volumetric dataset. Each type of 3D microscopy comes with its own mechanical and optical artifacts that require modality-specific attention to achieve precise 3D stitching. Therefore, stitching algorithms have evolved over time to meet the particular needs of different imaging modalities particularly when it comes to managing increasingly large data sets. This section will discuss currently available tools for block-face and SPIM image processing.

The main challenge in developing an accurate stitching algorithm is accounting for different types of distortion in the X, Y, and Z directions. For example, manual histological sectioning is generally uniform in section thickness (Z direction), but mounting tissue on glass slides often introduces non-uniform distortion along the X-Y axes. In mapping through manual serial sectioning, it is typical to use stitching software provided with the microscope to perform X-Y stitching but then an in-house method to align the data along the Z-axis for 3D registration (Hintiryan et al., 2016; Bienkowski et al., 2018). Although block-face imaging also relies on sectioning, the X-Y-Z distortion is minimal due to its unique imaging configuration as explained in section 2.1 and Figure 3. Therefore, stitching modules in Fiji (or ImageJ) can be used to perform repeated two-dimensional stitching throughout the entire image volume (Ragan et al., 2012). To mitigate tile line artifacts in 2D reconstruction, image tiles are normalized by an average Z projection (Ragan et al., 2012). Then, corrected tiles can be aligned using cross-correlation, and the overlapping pixels can be blended using linear averaging (Ragan et al., 2012). Because this pipeline is accomplished in Fiji, it is free, and little programming skill is needed to stitch data. However, this simple Fiji stitching utilizes high amounts of memory, making it less suitable for larger data sets (e.g., over 1 TB). This has been recently overcome by TeraStitcher, a python- based code, that uses meta-data stored in the acquired images to calculate the minimum number of stitching lines needed reducing the computational costs of stitching large data (Bria and Iannello, 2012; Hörl et al., 2019; Kirst et al., 2020). TeraStitcher also simultaneously performs illumination corrections and allows the user to manually correct stitching misplacement (Bria and Iannello, 2012). BigStitcher utilizes a similar method to reduce the computational costs of stitching and is available in Fiji as a plugin (Hörl et al., 2019). However, BigStitcher also incorporates iterative minimization of square displacements to improve poor distance control between disconnected objects and improved correction for spherical and chromatic aberrations (Matsuda et al., 2018; Hörl et al., 2019). Spherical aberrations arise from light that enters a lens at the edge of the lens and gets focused on a different point in the tissue compared to the lights entering the center of the lens making the edges of an image where stitching occurs appear blurred which impedes accurate stitching and further analysis (Diel et al., 2020). Chromatic aberrations occur because the refractive index and the focal length of a lens are dependent on wavelength. Therefore, different wavelengths of light will produce shifted images. Thus, spherical and chromatic aberration correction can significantly improve the alignment of multichannel images (Marimont and Wandell, 1994; Matsuda et al., 2018).

SPIM datasets also contain an additional aberration because excitation and emission light need to go through the whole cleared 3D volume which introduces optical aberrations in all 3D axes including the z-direction (Kirst et al., 2020). WobblyStitcher is a python-based tool that accounts for this 3D aberration by creating max z projections to calculate a z profile per sample which is then combined with a global optimizer to complete tile merging (Kirst et al., 2020). While this stitching method solves an important problem for processing SPIM images, WobblyStitcher is only available as a python code requiring some programming skill to handle while also demanding significant computational power and time (Kirst et al., 2020). Another artifact that is unique to SPIM is striping which is caused when any object blocks light penetration into the sample. Destriping tools have been developed to remove striping and other shadows by applying a low pass Fourier transform filter on the 2D wavelet transform (Swaney et al., 2019; Kirst et al., 2020). This prevents the removal of important biological information such as entire vessels/axons that align with the artifact while smoothing ripple-like striping. Many other denoising algorithms are also available to enhance the signal to noise ratio but should be approached with caution to prevent the introduction of unexpected artifacts (Tyson and Margrie, 2021).

### 4. Automated Signal Detection

Cellular resolution information in the whole brain makes manual detection and counting difficult if not impossible. To overcome this issue, many automated cell counting and tracing tools have been developed. They can be divided into three large categories: Filter-based, Machine Learning (ML), and Deep Learning (DL).

**Figure 4:**
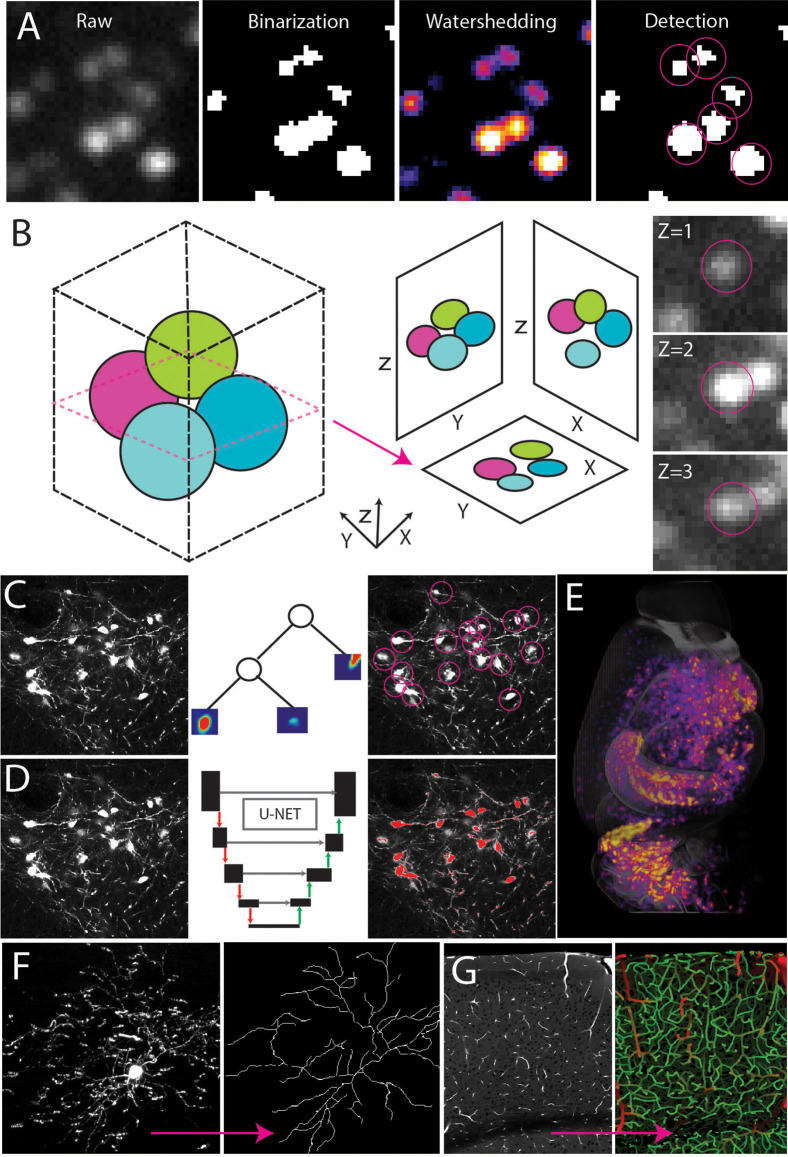
Automated signal detection.** (A)** Cell detection pipeline using filter-based methods including intensity filters and water-shedding. **(B)** Cells can be captured in multiple planes making it necessary to identify the connected components of a central point in the sphere. **(C–E)** Random forest-based machine learning **(C)** or deep learning segmentation **(D)** to detect cell type signals across the whole brain **(E)**. **(F,G)** Signal detection and tracing to achieve full 3D reconstruction of a single neuron (**F**, adapted from Janelia MouseLight NeuronBrowser) or cerebrovascular network analysis **(G)**.

Filter-based, non-machine learning detection utilizes simple thresholds to select features and define objects (Sbalzarini, 2016). Detection often starts with binarization or masking through intensity thresholds (Figure 4A). Following binarization, objects in the image can be separated using other filters such as water-shedding, fast-marching, erosion, size, circularity, and many more refined methods (Figure 4A). These simple methods can be applied to brain mapping samples, but special attention must be paid to the three-dimensional nature of the imaging as objects may appear in multiple planes depending on the resolution (Figure 4B). ClearMap is a tool that identifies the local maximum of a 3D spherical object and assigns a single coordinate to the center of a sphere with all connected pixels belonging to that cell/coordinate which prevents overcounting of cells appearing in multiple planes (Renier et al., 2016). This filter-based detection is simple and easily achievable in any image processing software but has limited functionality because each algorithm must be designed per experiment and tuned by a user which can lead to highly subjective selection patterns.

Because filter-based detection is not flexible, it does not lend itself to complex or densely packed structures with variable intensity or poor signal to noise ratio (Shamir et al., 2010; Sbalzarini, 2016). For example, simple thresholding in images with an inherent intensity gradient and multiple structures of interest is tedious and subjective as each ROI in a large 3D brain may have a unique intensity profile (Renier et al., 2016; Mergenthaler et al., 2021). A task such as this (e.g., without clear-cut rules and requiring repeated efforts) is better tackled with machine learning approaches which can do any number of tasks with adaptive thresholding (Figure 4C). Ilastik is a popular user-friendly software that will utilize machine learning to segment data based on a small number of user inputs and training (Renier et al., 2016; Berg et al., 2019; Kreshuk and Zhang, 2019). Ilastik mainly uses random forest clustering to calculate probabilities based on experimenter inputs to determine detected signals (Renier et al., 2016; Berg et al., 2019; Kreshuk and Zhang, 2019). The Weka plug-in for Fiji also works in a similar manner to Ilastik, making it a useful add-on to Fiji-based analysis pipelines (Arganda-Carreras et al., 2017).

Though machine learning is more flexible than filtering methods the quality of detection depends on the quality of the training set and the selection criteria making it subject to human bias and limited to concrete mathematical-based problems (Sbalzarini, 2016). When feature selection is more challenging, deep learning (DL) can provide better automatic annotation (Yangt et al., 2017; Figure 4D). For example, a set of morphologically distinct neurons labeled with the same fluorescent color in the same brain can still be separated with DL which uses human annotation to develop its own criteria/ algorithm for counting and classifying structures (Kim et al., 2015, 2017; Ning et al., 2020; Tyson et al., 2021; Wu et al., 2021; Zhang et al., 2021; Figure 4D). DL requires much more user input than machine learning, but it allows the neural network more freedom in algorithm development, however, implementing DL requires more computational skill and power than other methods making it more challenging to use for scientists with a basic level of computational skill. Finally, none of these automated detection algorithms are 100% accurate, so quality control and mathematical validation are critical.

The main applications of automated annotation include cell/synapse counting, connectivity analysis, and single cell morphological reconstruction. Counting cells (e.g., cell nuclei) is relatively simple due to their uniform shape and is often done using filter-based counting (Renier et al., 2016; Asan et al., 2021; Figure 4E). In fact, many ML and DL cell counting approaches will start with filter-based output and perform additional refinements (Kim et al., 2015; Renier et al., 2016; Newmaster et al., 2020; Wu et al., 2021). If the labeling is cytosolic, the DL based NeuroGPS tool may be employed to isolate the soma from the axon and dendritic tree (Quan et al., 2013; Figure 4D). The benefit of this approach is that it can be combined with more advanced connectivity and morphology analyses such as single cell reconstruction and tracing (Gong et al., 2013; Zheng et al., 2013; Economo et al., 2016, 2019; Han et al., 2018; Winnubst et al., 2019; Gouwens et al., 2020; Xu et al., 2021; Figures 4F,G). Single cell tracing can be accomplished in programs such as Allen’s Vaa3D which contains plug-ins utilizing Euclidean distance to identify the connected components of a skeletonized image (Peng et al., 2010; Friedmann et al., 2020). Other Vaa3D plug-ins such as Deep Neuron utilize deep learning to take tracing to the next level by annotating axons and dendrites as separate compartments within a single image (Zhou et al., 2018). Though simple filter-based methods have been used to quantify the density of axons in tracing experiments, these methods cannot assign a single axon to a single soma in images with densely packed structures. TrailMap and MIRACL use DL to trace back overlapping axons to their respective soma adding another level to connectivity analysis (Çiçek et al., 2016; Gerfen et al., 2018; Goubran et al., 2019; Friedmann et al., 2020; Li and Shen, 2020). In neural connectivity analysis, another interesting challenge is quantifying synapses as the size of synaptic puncta resides near the limit of light diffraction and synapses are densely packed along neural processes. To address this issue, ML and DL were again employed allowing researchers to map synapses across large three-dimensional regions, yet no study to date has looked at the whole brain completely due to the daunting data size and magnitude of the required analysis (Zhu et al., 2018; Cizeron et al., 2020; Curran et al., 2021).

Many of these algorithms can be modified and applied to other cell types within the nervous system as well. For example, tracing the cerebrovasculature has become a popular new frontier in 3D brain mapping as the densely packed microvessels of the brain and their connectivity patterns are deeply intertwined with neural structures (Blinder et al., 2013; Xiong et al., 2017; Schmid et al., 2019; Ji et al., 2021; Wu et al., 2021; Figure 4G). This complex system also presents a unique challenge requiring higher resolution imaging, precise image reconstruction, and connectivity analysis (Xiong et al., 2017; Kirst et al., 2020; Todorov et al., 2020). Recently, open source programs developed based on machine learning have been used to trace and skeletonize vasculature throughout the brain (Kirst et al., 2020; Todorov et al., 2020; Wu et al., 2021).

In summary, recently developed open source software and AI-based tools provide great opportunities to perform high throughput detection of various cell type features in high- resolution 3D datasets. As the focus of brain mapping expands beyond neurons, many new algorithms will become available to better analyze different cell type densities and morphologies.

### 5. Mapping Signals onto Standard Spatial Framework

**Figure 5:**
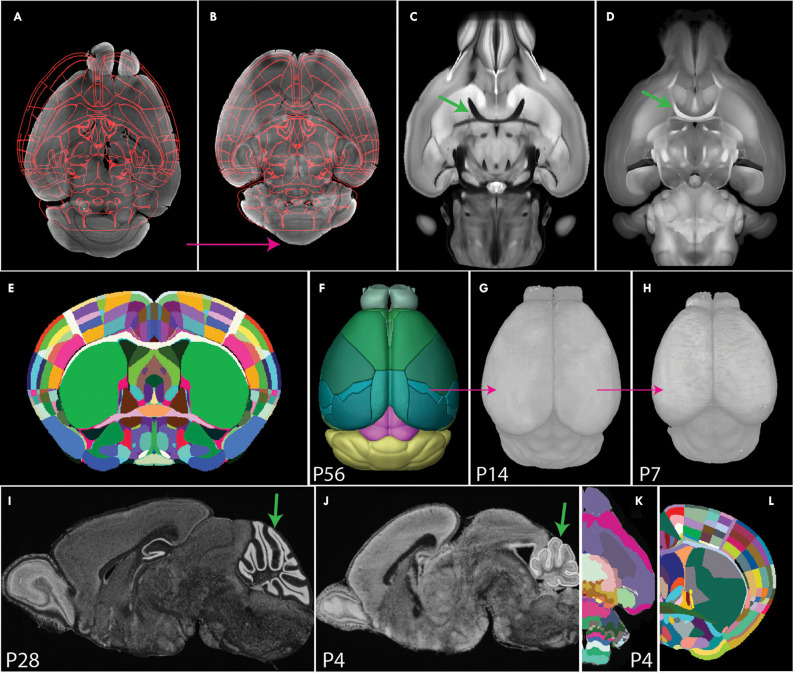
Image registration to a common coordinate framework.** (A,B)** Image registration transforms individual samples to align with an annotated template atlas **(A)**, allowing detected signals to be assigned to anatomical labels **(B)**. **(C,D)** Comparison of STPT **(C)** and SPIM **(D)** images. The STPT image has a characteristic intensity profile with dark white matter tracts (arrow in **C**), while SPIM has light white matter tracts (arrow in **D**). **(E,F)** The Allen CCF was generated by averaging many STPT images of adult mouse brains **(E)** and was annotated with smooth 3D labels **(F)**. **(G,H)** 3D Developmental mouse brain atlases at postnatal day 14 **(G)** and 7 **(H)** based on STPT imaging *via* back registration and label importation. **(I,J)** Nissl staining sagittal image of P28 **(I)** and P4 mouse brain. Arrows highlight morphological differences in the cerebellum between the two ages. **(K)** P4 3D Developmental atlases derived from 2D annotations. **(L)** Franklin-Paxinos labels mapped onto the Allen CCF template with detailed segmentations in the dorsal striatum.

Cell type signals from individual brains vary in quantity and spatial distribution. Moreover, each individual brain and its sub-regions can have different volumes and shapes (Toga and Thompson, 2001; Allen et al., 2008; Shimono, 2013; Janke and Ullmann, 2015; Lee et al., 2021). Therefore, reproducible analysis and interpretation of whole brain 3D data from multiple samples requires mapping onto a common coordinate framework. By aligning or registering individual samples onto a standardized annotated template brain (atlas), identified signals can be automatically assigned to anatomical areas in an unbiased way (Figure 5A). Moreover, using this spatial framework and mathematical deformation allows ROI volume to be calculated from each brain for more precise estimates of cell density per area (Kim et al., 2015, 2017; Newmaster et al., 2020). Here, we will discuss image registration tools and currently available atlas frameworks.

#### 5.1. Image Registration

The process of aligning one image to another (e.g., registration) utilizes rigid, affine, and other non-linear transformations in varying combinations and implementations (e.g., algorithms) to achieve optimal image matching (Toga and Thompson, 2001; Klein et al., 2010; Avants et al., 2011, 2014; Beare et al., 2018; Balakrishnan et al., 2019; Figures 5A,B). Rigid registration is restricted to translation and rotation, while affine transformation allows scaling and sheering along all three axes with each pixel/voxel undergoing an identical transformation (Holden, 2008; Song et al., 2017). Other non-linear registration including elastic and diffeomorphic registration allows deformable rigid, scaling, and sheering to be applied to different parts of an image (Avants et al., 2008, 2011; Klein et al., 2010; Mansilla et al., 2020; Sun and Simon, 2021). Diffeomorphic registration has the most freedom as each pixel or voxel can be moved independently which can increase registration quality, but it is computationally expensive compared to rigid and affine registration methods (Avants et al., 2008, 2011; Lee et al., 2021). Therefore, the best alignment method combines these methods in a way that maximizes accuracy while minimizing computational time. Alignment accuracy can be evaluated by mathematical procedures that calculate how well intensity values align with common metrics such as cross correlation which maximizes the correlation between normalized intensity values and mutual information which matches features such as edges or intensity gradients (Andronache et al., 2008; Jiang et al., 2017; Bashiri et al., 2018). These methods can be further explored for specific use cases in Avants et al., 2011.

Preprocessing steps and registration can be performed using various open source programs like Elastix, Advanced Normalization Tools (ANTs), Insight Toolkit (ITK), and the Statistical Parametric Mapping (SPM) package (Avants et al., 2008, 2014; Klein et al., 2010; Kronman et al., 2020). Elastix is a Unix command line interface that efficiently applies rigid, affine, and elastic registration. This program has been used to register data to reference atlases and co-register labels between two differing adult mouse brain atlases (Ragan et al., 2012; Kim et al., 2015; Renier et al., 2016; Chon et al., 2019; Kirst et al., 2020; Newmaster et al., 2020; Son et al., 2020; Wu et al., 2021). ANTs is also a Unix interface based on registration tools from ITK which houses all of the described registration methods (Ibanez et al., 2002; Avants et al., 2014). ITK has been used to create mouse brain templates, register STPT samples to the Allen common coordinate framework, and perform a number of human studies making it broadly applicable in 3D image analysis (Kuan et al., 2015; Pichet Binette et al., 2021; Tustison et al., 2014; Wang et al., 2020; Whitesell et al., 2021). The SPM package, housed within MATLAB, is commonly used for co-registering and analyzing MRI data in human brain mapping studies including mapping of functional resting state networks (Friston et al., 2007; Kronman et al., 2020). Additional programs for registration include FSL (Jenkinson et al., 2012), AFNI (Cox, 1996), Fiji (Schindelin et al., 2012), Medical Image Registration Toolkit (Rueckert et al., 1999; Schnabel et al., 2001), and FreeSurfer (Reuter et al., 2010). Many of these packages have been wrapped together in command line or graphical user interface (GUI) tools for an easier user experience. For example, Nipype, an open source python tool, integrates ANTs, AFNI, SPM, FSL, and more, while BrainMaker and NeuroInfo, a pair of commercial packages, combine ITK and additional custom software in a GUI to perform image preprocessing and registration (Tappan et al., 2019). Most software is compatible with different 3D imaging data including human neuroimaging. However not all software is universally applicable across species, so additional plugins have been developed for commonly used preclinical models, such as SPMMouse, which allow SPM to be used with mouse brain data (Sawiak et al., 2009). A more comprehensive list of available software can be found at www.nitrc.org.

A major conundrum in the imaging field is cross-modality registration because current registration methods rely on intensity contrast to guide alignment. For example, STPT and LSFM imaging with iDISCO based cleared samples have different contrast profiles such as fiber tracks being labeled as dark and light contrast, respectively (Figures 5C,D, arrows). Therefore, attempting to align light and dark regions between images may result in erroneous registration. One solution is to use DL networks trained by expertly aligned multimodal data to register novel data (Fu et al., 2020). Many open source packages are presently available to explore DL approaches to medical image registration, and improving DL registration will increase the quality and applicability of image registration as image resolution rises and whole brain imaging of larger primates, including humans, evolves (Yangt et al., 2017; Balakrishnan et al., 2019; Mok and Chung, 2020; Wang and Zhang, 2020). Future efforts in neuroimaging will require innovative neuro-engineering solutions to reduce the computational resources and time required to register large images. Further information about image registration can be found in the following review articles: (Viergever et al., 2001, 2016; Hess et al., 2018; Fu et al., 2020).

#### 5.2. Atlases as Common Spatial Framework

An atlas defines anatomical boundaries that can be used to interpret target signals in an anatomical context. Historically, brain atlases were manually annotated on 2D images by expert neuroanatomists based on histological features. These atlases exist for many species including mice (Kaufman, 1992; Thompson et al., 2014; Paxinos and Franklin, 2019), rats (Paxinos and Watson, 2006), non-human primates (Paxinos et al., 2000; Palazzi and Bordier, 2008), and humans (Mai et al., 2008; Ding et al., 2016). However, limitations of sparse labeling, uni-planar views, and tissue distortion render these atlases sub-optimal for application to 3D brain mapping. Today, 3D digital brain atlases are available in a few selected species including humans (Mazziotta et al., 2001; Amunts et al., 2020) non-human primates (Liu et al., 2018; Woodward et al., 2018), and mice (Dorr et al., 2008; Chuang et al., 2011; Szulc et al., 2015; Young et al., 2021). Unfortunately, many of these atlases are often based on only one sample, lack detailed segmentation, and/or are subject to modality-specific tissue distortion. The state-of-the-art atlas used for localizing 3D whole mouse brain signals is the Allen common coordinate framework (CCF), an STPT based atlas comprised of 1,675 samples averaged together with highly detailed and smoothed 3D labels (Wang et al., 2020; Figures 5C,E,F).

In contrast to the detailed Allen CCF for the adult brain, 3D CCFs for neurodevelopmental stages are scarce which presents a major hurdle to understanding the developmental trajectories of different cell types in normal and pathological conditions (Puelles et al., 2013; Levitt and Veenstra-VanderWeele, 2015). Though registration tools are applicable at any age, they require size and shape matching templates. To generate such templates for early post-natal brain mapping, we previously created intensity averaged STPT brain templates by registering more than 10 samples onto one best brain at each postnatal day (P) 7, 14, 21, and 28. These 3D templates were annotated by back registering the Allen CCF 3D labels to each template in sequence from oldest to youngest using Elastix. These brains were successfully used to quantify oxytocin receptor cell density across the whole mouse brain in development (Newmaster et al., 2020; Figures 5F–H, pink arrows show back registration). While back registration is a common approach to developmental brain mapping, it is not the best approach as ROIs may undergo differential rates of development depending on their embryonic origin (Zhang et al., 2005; Chuang et al., 2011; Figures 5I,J, arrows for the cerebellum). A more biologically sound method for creating developmental atlases is to create age-specific digitized templates or reference brains for developmental time points with anatomical labels based on neurodevelopmental perspective (Puelles and Ferran, 2012; Puelles et al., 2013; Thompson et al., 2014). Recent advances have attempted to convert 2D developmental atlases to 3D atlases, but these converted 3D anatomical labels still contain jagged surfaces due to interpolation errors (Young et al., 2021; Figure 5K). Therefore, neuroscience is still in great need of accurately annotated 3D developmental atlases that can be applied to seamlessly map and interpret datasets from different embryonic/early postnatal ages and imaging modalities.

Current 3D atlases also lack cross-modality capabilities. The current gold standard, the Allen CCF, is obtained from STPT images which does not align well with datasets from other imaging modalities (e.g., light-sheet fluorescence microscopy). To address this gap, a light-sheet optimized template brain was developed by averaging light-sheet images and importing Allen labels to this space (Perens et al., 2021). However, such a template is likely to have volume distortion specific to the chosen tissue clearing method. Thus, the ideal spatial template should come from an imaging modality with minimal distortion such as high resolution, in skull, *ex vivo* MRI. Moving forward, future atlases should be based on distortion-free template space while accommodating multiple imaging modalities, allowing for cross-modality comparison of datasets from any experimental setup.

### 6. Data Sharing and Federation

Whole brain mapping data contains rich anatomical and functional data that one single publication cannot capture. Thus, data sharing has become increasingly important to facilitate further analysis of these hard-earned datasets. The FAIR data principles describe guidelines for making digital data findable, accessible, interoperable, and reusable (Wilkinson et al., 2016). The FAIR principles focus on the proper use of metadata to describe digital datasets in a way that is readable by both computers and human scientists optimizing collaboration and data reuse. The International Neuroinformatics Coordinating Facility (INCF) has endorsed the use of the FAIR principles and added more specifications (Abrams et al., 2021). For instance, in the United States, all microscopy data collected as part of a BRAIN Initiative grant must be shared *via* the Brain Imaging Library (BIL; Benninger et al., 2020). The BIL will soon require a list of essential metadata for 3D BRAIN Microcopy for uploaded datasets including data contributors, descriptors, funders, instruments, images, specimens, and related publications (Ropelewski et al., 2021). Data from omics experiments, electrophysiology, MRI, and electron microscopy also have their own respective archives as described by the NIH Notice of Data Sharing Policy for the BRAIN Initiative allowing this data to be combined with imaging (National Institutes of Health, 2019). The Human Brain Project, EBRAINS, and INCF all lay out similar requirements across the world (Kleven and Bjerke, 2021). In addition to public databases, web visualization has been a useful data sharing tool that avoids the need to download large-scale data. For instance, we created a website^4^ where high-resolution datasets of oxytocin receptor expression, the oxytocin neuron wiring diagram, and full brain vasculature can be easily visualized. Allen Institute of Brain Sciences also has a large repository of brain data on the Allen Brain Map website^5^.

With the huge influx of large-scale mapping datasets, scientists need to consider how to confederate different data from individual research groups for integrative analysis. The main barrier to developing a brain mapping community with highly reproducible and shareable data lies in the existence of multiple atlases with conflicting segmentation and ontology (Chon et al., 2019; Figure 5L). As mentioned before, comparison between different datasets requires registration into a standardized common coordinate framework with a coherent anatomical label system like the Allen CCFv3 (Muñoz-Castañeda et al., 2020; Wang et al., 2020; BRAIN Initiative Cell Census Network (BICCN), 2021; Takata et al., 2021). To further facilitate data confederation, there is a significant need to have a standardized anatomical nomenclature system within and across species. Anatomical labels are subject to how different anatomists select delineation criteria from their knowledge, so putting different anatomical systems in a single common space can allow neuroscientists to interpret their data in a more streamlined and coherent way. For example, we recently integrated Franklin-Paxinos atlas labels onto the Allen CCF and added detailed segmentation in the dorsal striatum (Chon et al., 2019). This way, data can be mapped onto the Allen CCF, but users can choose different atlas labels to interpret their data.

Finally, as brain mapping advances, standardized neuroanatomy terminology across species will be critical to cross-species comparison and evolutionary studies. The Neuroscience Lexicon^6^ and the Uberon ontologies database^7^ were developed as resources to track neuroanatomical structure names and concepts, incorporating them into a structured knowledge management framework with unique identifiers for each concept (Larson and Martone, 2013; Haendel et al., 2014; Miller et al., 2020; Yuste et al., 2020). Standardized anatomical labels based on current data will facilitate translational research by providing a common language between clinical and preclinical work. In summary, data standardization and confederation can unlock the true power of community-driven neuroscience approaches to gain a comprehensive understanding of brain organization in many species.

## Conclusion

Whole brain mapping at the cellular resolution is becoming increasingly popular and important to gain a better understanding of cellular organizational principles across the whole brain. When done correctly, we can see the cellular composition and arborization across many brain regions, in essence allowing us to see both the individual “trees” within the context of the “forest”. However, brain mapping requires optimization at multiple steps with experiment-specific considerations. Our review provides basic information on each of these steps and highlights major considerations and resources to facilitate the optimization of brain mapping experiments. Finally, making mapping data more widely accessible will facilitate a new era in neuroscience where collaboration and data sharing can provide enormous synergy and impact to facilitate the advance of neuroscience research.

## Author Contributions

KTN and YK conceptualized the manuscript. KTN wrote the initial manuscript draft and figures and developed the manuscript further with other authors (FAK, YW, YK). YK handled the funding and critically revised the manuscript. All authors contributed to the article and approved the submitted version.

## Conflict of Interest

The authors declare that the research was conducted in the absence of any commercial or financial relationships that could be construed as a potential conflict of interest.

## Publisher’s Note

All claims expressed in this article are solely those of the authors and do not necessarily represent those of their affiliated organizations, or those of the publisher, the editors and the reviewers. Any product that may be evaluated in this article, or claim that may be made by its manufacturer, is not guaranteed or endorsed by the publisher.

## Footnotes

^1^ https://connectivity.brain-map.org/transgenic

^2^ http://www.gensat.org

^3^ https://neuroscienceblueprint.nih.gov/resources-tools/blueprint-resources-tools-library/cre-driver-network

^4^ https://kimlab.io/

^5^ https://portal.brain-map.org/

^6^ https://scicrunch.org/scicrunch/interlex/dashboard

^7^ https://www.ebi.ac.uk/ols/ontologies/uberon
